# Investigating the Influence of Virtual Human Entourage Elements on Distance Judgments in Virtual Architectural Interiors

**DOI:** 10.3389/frobt.2019.00044

**Published:** 2019-06-28

**Authors:** Sahar Aseeri, Karla Paraiso, Victoria Interrante

**Affiliations:** ^1^Department of Computer Science and Engineering, University of Minnesota Twin Cities, Minneapolis, MN, United States; ^2^Department of Computer Science and Engineering, Arizona State University, Tempe, AZ, United States

**Keywords:** virtual environments, entourage elements, distance perception, virtual human, photo-realistic avatar

## Abstract

Architectural design drawings commonly include *entourage elements*: accessory objects, such as people, plants, furniture, etc., that can help to provide a sense of the scale of the depicted structure and “bring the drawings to life” by illustrating typical usage scenarios. In this paper, we describe two experiments that explore the extent to which adding a photo-realistic, three-dimensional model of a familiar person as an entourage element in a virtual architectural model might help to address the classical problem of distance underestimation in these environments. In our first experiment, we found no significant differences in participants' distance perception accuracy in a semi-realistic virtual hallway model in the presence of a static or animated figure of a familiar virtual human, compared to their perception of distances in a hallway model in which no virtual human appeared. In our second experiment, we found no significant differences in distance estimation accuracy in a virtual environment in the presence of a moderately larger-than-life or smaller-than-life virtual human entourage model than when a right-sized virtual human model was used. The results of these two experiments suggest that virtual human entourage has limited potential to influence peoples' sense of the scale of an indoor space, and that simply adding entourage, even including an exact-scale model of a familiar person, will not, on its own, directly evoke more accurate egocentric distance judgments in VR.

## 1. Introduction

This paper represents an extended version of work that was previously published (Paraiso and Interrante, 2017) in the Proceedings of EuroVR 2017. The previously un-published research contributions are limited to the second experiment.

In the field of architectural design, immersive virtual reality (VR) technology offers clients the ability to experience proposed structures from a first-person perspective before they are built, potentially enabling them to make better-informed decisions about critical features such as room size, ceiling height, etc. However, human-subjects experiments have consistently found systematic biases in peoples' judgments of egocentric distances in immersive virtual environments, resulting in an average underestimation of ~25% in head-mounted-display-based VR as compared with similar distance judgments in the real world (Renner et al., 2013). This has serious implications for a reliance on VR in architectural design reviews, as such errors could, for example, make a 9-foot ceiling feel more like an 8-foot ceiling, or a 12′ × 15′ room feel more like a 10′ × 12′ space. While there are some indications that the newest generation of lighter, more ergonomic and wider-field-of-view head-mounted displays (HMDs) may afford more accurate distance judgments than HMDs of the past (e.g., Young et al., 2014; Creem-Regehr et al., 2015), the use of these new displays does not, on its own, appear to eliminate the VR distance underestimation problem completely (Kelly et al., 2017). Additionally, although numerous work-arounds have already been proposed for addressing the distance underestimation phenomenon in HMD-based VR, each of those methods has some shortcomings, making the possibility of using virtual human (VH) entourage elements as a mitigating intervention for this problem a potentially attractive alternative.

In this paper, we describe two experiments that explore the potential of using photorealistic virtual human entourage elements to improve distance perception accuracy in HMD-based immersive virtual environments. In each experiment, we use a photorealistic virtual model of a real person who is known to the participants. In our first experiment, which was previously presented at EuroVR 2017 (Paraiso and Interrante, 2017), we assessed the potential impact of augmenting a virtual indoor environment with a static or animated entourage agent. To extend that work for this Special Topics issue featuring Best Papers from EuroVR 2017, we conducted a second, complementary experiment, in which we vary the scale of the familiar virtual human model to more broadly explore the scope of the potential impact of virtual human entourage on spatial perception in virtual architectural environments.

## 2. Previous Work

Previously proposed methods for addressing the distance underestimation phenomenon in HMD-based VR include: (1) enforcing an acclimation/training period during which people receive various types of feedback (e.g., visual, proprioceptive, haptic, etc.) about the mapping between what they see in VR and how far away things actually are when they physically try to reach or walk toward them (Richardson and Waller, 2007; Kelly et al., 2014); (2) initiating the VR experience in a photorealistic replica of the user's concurrently-occupied real physical environment (Steinicke et al., 2009); (3) rendering the virtual environment using an artificially exaggerated geometric field of view (gFOV), effectively applying a global “minification” to the user's view of their surroundings (Li et al., 2015); (4) rendering the virtual environment from position that is systematically lower than the user's actual eye height (Leyrer et al., 2015); (5) introducing a bright light into the periphery of the user's visual field (Jones et al., 2013); or (6) embodying the user in a virtual self-avatar (Ries et al., 2008; Mohler et al., 2010). While each of these proposed interventions has shown some success, each also has some limitations. For instance: re-calibrating one's distance perception by physically walking with feedback may not generalize from shorter to longer distances (Kelly et al., 2014), or to distances in directions that do not afford walking, such as ceiling height judgments. In addition, clients may not want to have to go through acclimatization exercises prior to viewing a model, and if acclimitization occurs during the course of viewing the model of interest, it is possible that clients could form erroneous first-impressions about the suitability of a space that might be enduring even after re-calibration has been achieved. A concern with the use of distortion-based interventions such as view minification or eye height perturbation is the assumption that a constant amount of correction is appropriate. Besides the challenge of accounting for individual differences in the amount of distortion needed to evoke accurate distance judgments, there the problem that as adaptation or re-calibration occurs during the immersive experience, the intentionally-corrective distortion might over time inadvertently become counter-productive. Finally, while the use of self-avatars is an attractive possibility, any intervention that requires extra equipment or encumbrance may not be well-received by users. Our architectural colleagues have found that clients can sometimes be hesitant even to want to put on an HMD; such users are unlikely to want to wear a full body motion-capture suit, or even the minimal set of auxiliary sensors that would be needed to approximately animate a reasonable self-avatar via inverse kinematics. Overall, therefore, while many potential solutions do already exist for the problem of distance underestimation in VR, we feel that the exploration of additional solutions remains a worthwhile endeavor.

Architects have a long history of including small human figures in their drawings to indicate scale and to show how the space might look while in use (Anderson, 2002; Colonnese, 2017). Our research is inspired by the possibility that adding such figures to building models in VR might similarly facilitate more accurate spatial understanding in those environments. As such interventions have not been previously explored, we feel that it is appropriate to assess how well they might work and to try to elucidate the theory behind their potential effect.

With regard to entourage, we have found in previous work that augmenting virtual environments with ergonomically size-constrained familiar inanimate objects, such as chairs, does not evoke more accurate judgments of egocentric distance in those environments (Interrante et al., 2008). Nevertheless, the importance of using representatively-furnished models of virtual architectural interiors is supported by real-world studies showing that furnishings can have a significant impact on spaciousness judgments in an architectural context: for example, in one experiment architecture students judged a real room to be significantly less spacious when it was either empty or over-crowded than when it was moderately furnished (Imamoglu, 1973).

With regard to virtual humans, prior research has shown that enabling people to see a virtual representation of their own body can lead to more accurate judgments of 3D distances in VR (Ries et al., 2008; Mohler et al., 2010), but it is not clear to what extent representations of other people, as opposed to other familiar objects, would be advantageous for conveying metric size. Human height can vary relatively widely between different individuals (NCD, 2016), so it seems unlikely that an unfamiliar human body could serve as a reliable measure of scale. However, studies have shown that people are remarkably adept at estimating the height of an unknown person from a full-length photograph (Kato and Higashiyama, 1998), and it has been observed that people tend to pay particular attention to human figures (as opposed to inanimate objects) when viewing images (Judd et al., 2009), which may reflect social requisites (Risko et al., 2012). Furthermore, it is likely that implicit assumptions of size constancy are stronger with respect to humans than to familiar inanimate objects, due to ecological constraints: if one sees a door that is only half as tall as a person standing next to it, it is much more likely that the door is unusually small than that the person is unusually large.

With regard to expected similarities and differences in the impact of seeing one's own body in VR vs. seeing the bodies of others, several points bear consideration. First is the theory behind why people are able to more accurately estimate egocentric distances when they are embodied in a first-person (Ries et al., 2008) or third-person (Mohler et al., 2010) self-avatar. On the one hand, it could be that embodiment evokes a more visceral sense of presence in the virtual environment (Interante et al., 2005) or that it makes the affordances for action in the virtual world more explicit (Geuss et al., 2010). On the other hand, several studies also suggest that people may use the apparent size of their own body in VR to scale their perception of their surrounding environment. Specifically, studies have shown that when people are embodied in a first-person avatar in proximity to an object with which there is an affordance for interaction using a virtual body part (e.g., hand or foot), their perception of the nearby object's size is affected by the apparent size of their virtual avatar's relevant body part. For example, Jun et al. (2015) found that people judge gaps on the ground to be wider when their self-avatar consists of a pair of smaller-sized disembodied feet, and Linkenauger et al. (2013) found that people perceive generic objects on a table to be smaller when they are viewed in the context of a self-avatar that has a larger virtual hand. However, knowing the impact of a self-avatar only partially informs the impact of virtual others. In the same study where they found a self-avatar scaling effect, Linkenauger et al. (2013) also found that people did not scale their perception of the size of a generic object to the apparent size of another avatar's hands. However, Langbehn et al. (2016) found that when people were asked to differentiate between being a virtual giant (or midget) in a right-sized virtual environment vs. being right-sized in a miniature (or gigantic) virtual environment, their answers *were* affected by the apparent sizes of other co-located collaborators whose virtual bodies they could see: people tended to always assume that their colleagues' avatars were right-sized, regardless of their own perspective, and to adjust their sense of the scale of their surrounding virtual environment accordingly. For example, when their colleagues appeared small with respect to the surrounding virtual environment, people were more likely to adopt the interpretation that the virtual environment was gigantic. Previous work therefore leaves some room for further elucidation of how the virtual body sizes of others might affect one's own sense of the scale of a co-occupied space, or of egocentric distances within it.

Finally, most pertinent to our current investigations, Ragan et al. (2012) report an exploratory study in which people made more accurate spatial judgments in the context of a desktop virtual environment when non-realistic static or well-animated virtual characters were present in that environment than when no virtual characters or only badly-animated virtual characters were used. However, contemporary work by McManus et al. (2011) reported no significant improvement in peoples' egocentric distance perception accuracy in a realistically-rendered immersive virtual room environment when a generic dynamic autonomous agent was added.

It therefore remains an open question to what extent, and under what conditions, peoples' action-based judgments of egocentric distances in virtual architectural environments might be improved by the addition of a photorealistic static or animated virtual model of a familiar person. It seems plausible that a static model of virtual human of known actual height might serve as a more robust indicator of metric size than a generic VH model or an inanimate object. But it is unclear to what extent people might use the assumed size of that virtual human entourage model to calibrate their perception of size and distance in the shared virtual space, or to what extent people might feel a stronger sense of presence in a virtual environment when they experience it together with a realistically rendered model of a familiar person. It seems plausible that, in situations where a person cannot themselves be embodied in VR, the co-presence of a compellingly realistic independently dynamic virtual human agent might help evoke similar affordances for interaction with the virtual environment.

Our present paper seeks to extend existing insights from prior work through several novel manipulations. In our first experiment, we introduce the use of a photorealistic (rather than generic) virtual human model, whose size and appearance exactly match that of a person with whom the participant has just interacted, immediately prior to their immersion in VR. This intervention is designed to maximize the potential of the agent to serve as a reliable metric for absolute size judgments within the virtual environment (Ries et al., 2009). In our second experiment, we explore the impact of surreptitious modifications to the scale of the agent, to more broadly explore the scope of its potential effects.

## 3. Our Experiments

### 3.1. First Experiment

#### 3.1.1. Method

We used a within-subjects design to expose each participant, in different combinations, to three different conditions of virtual human presence—no VH, static VH, or dynamic VH—in three different virtual hallway environments, prior to having them make action-based egocentric distance judgments in those environments by walking without sight to previously-viewed target locations indicated by a virtual white mark at one of five different predefined distances on the virtual floor. Different hallway models were used in the different VH conditions to avoid that an impression of the interior space derived under one VH condition would influence distance judgments queried under a different VH condition. The assignment of virtual human condition to hallway environment, as well as the order of presentation of the different conditions was randomized between participants.

#### 3.1.2. Participants

We recruited a total of 18 participants (10 male, 8 female, ages 19–29, μ = 21.5 ±2.8) from our local University community via email lists and posted flyers. Participants were compensated with a $10 gift card to an online retailer. Our experiment was approved by our university's Institutional Review Board, and all participants gave written informed consent.

#### 3.1.3. Materials

**Figure 1:**
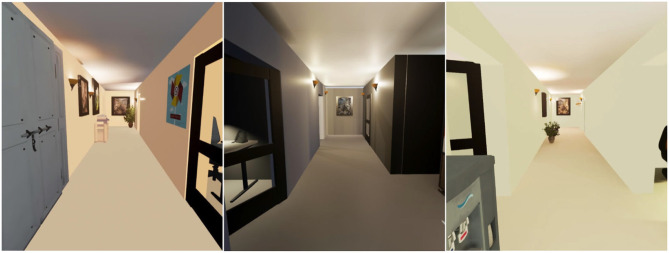
The three hallways models used in our experiment (Paraiso and Interrante, 2017).

We used Autodesk Maya to create three different virtual hallway models, which we imported into Unreal Engine, where they were lit and populated with system-provided assets such as light fixtures, picture frames, plants, doors, and windows, plus various items of furniture obtained from Arbitrary Studio^1^. The images in the paintings were obtained using a Google search for commercially-free-licensed images. We constructed the hallways so that all three models would be essentially structurally equivalent—each having the same length and width—but differing in appearance with respect to decorative details. Figure 1 shows what each hallway model looked like from the participant's starting position.

**Figure 2:**
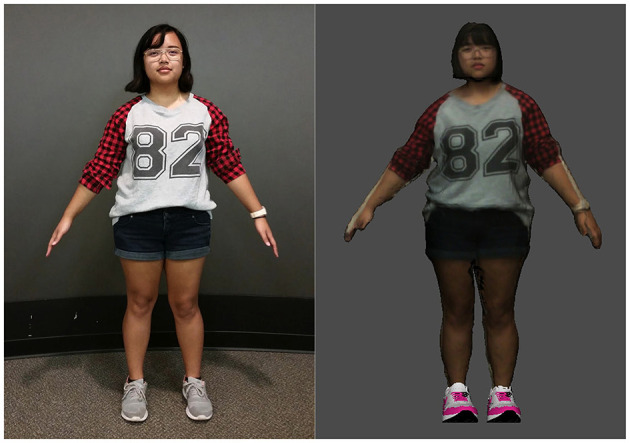
A photograph of the experimenter **(Left)** and her corresponding 3D model **(Right)** (Paraiso and Interrante, 2017).

We used the Skanect software by Occipital in conjunction with a Structure Sensor mounted on an iPad to capture a 3D model of the experimenter, which we imported into Mixamo to rig and animate. The experimenter wore the same outfit when conducting the study. The shoes were added to the model in a post-process (see Figure 2).

**Figure 3:**
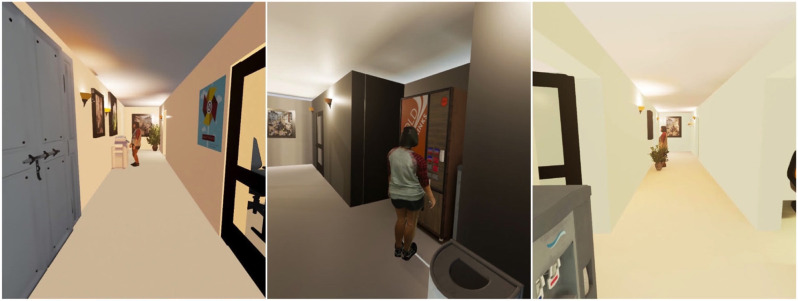
A view of the static virtual human in each of the three different virtual hallway environments (Paraiso and Interrante, 2017).

In each of the static agent conditions, the 3D virtual human model was placed in a visible position either beyond or to the side of the path over which the participant would need to walk in order to reach the targets used for the blind-walking distance judgments. In light of the findings of Jung et al. (2016), suggesting that people's estimates of distances to forward-facing virtual humans may be affected by social influences, we made sure to orient the model so that she was facing away from the participant and to position her so that her attention was implied to be engaged by some other item in the virtual hallway. Figure 3 shows what this looked like in each case.

**Figure 4:**
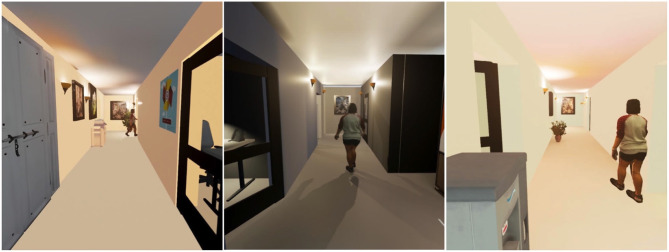
A representative view of the animated virtual human in each of the three different virtual hallway environments (Paraiso and Interrante, 2017).

For the animated agent conditions, we programmed the virtual human to traverse a path either across the virtual hallway or down the hallway in a direction away from the participant. In each case, the participant first had a view of the empty hallway before the virtual human walked into view. The agent appeared either from a doorway at the far end of the hall, from a doorway at the near end of the hall, or from behind the participant and to their right, and it exited from view through another doorway. We imported basic walking and turning motions from Mixamo to animate the agent's limbs, and used Unreal Engine's Animation Blueprint to define the character's movement through the scene. Figure 4 shows a representative frame from each of the three different character animations.

The experiment was conducted in our virtual reality laboratory, which approximately spans a 30′ × 29′ space. The virtual environment was rendered using Unreal Engine, running on an ORIGIN PC with an Intel Core i7 6850K Hex-Core 3.6 GHz processor, 32 GB DDR4 SDRAM (2,800 MHz), and a single 8GB NVIDIA GeForce GTX 1080 Founders Edition graphics card. Participants viewed the virtual environment using an HTC Vive head-mounted-display, which presents two 1080 × 1200 pixel resolution images on OLED displays, one for each eye, over a combined field of view of approximately 110° h × 100° v. The device weighs approximately 1 lb. and attaches securely to the head via wide elastic straps. 6DOF head tracking was accomplished using Valve's Lighthouse Tracking system, which spanned an approximately 15′ × 15′ area at one edge of the open lab space.

#### 3.1.4. Procedure

Participants were greeted at the door of our lab by the same experimenter who would be represented as a virtual human in the VR environment. Participants were scheduled by appointment so that each participant proceeded individually through the experiment, and no participant was exposed to any activity of any other participant. Each participant was first screened for adequate visual acuity, defined by the ability to successfully read lines of letters corresponding to 20/60 or above on a wall-mounted eye chart from a distance of 20′ without wearing glasses. We chose the distance of 20′ out of consideration of the focal distance of the optics in the HTC Vive, and participants were tested without glasses because they would not be able to wear their glasses in the HMD. None of the participants failed the visual acuity test. Next, participants were screened for stereo vision ability by asking them to identify two shapes of increasing depth complexity (a rectangle and a goldfish) presented as random dot stereo-grams on an Oculus Rift HMD. All of the participants passed the stereo vision test. After their eligibility to participate in the experiment had thus been verified, participants were given written instructions explaining the experiment procedure and were asked to sign an informed consent form. They then filled out a short survey providing basic demographic information (age, gender) and completed a baseline simulator sickness form (SSQ) (Kennedy et al., 1993).

Each participant was randomly assigned to a different set of three blocks of hallway/agent combinations, defined so as to ensure that each participant would be exposed to each hallway and each virtual human condition, and so that over the 18 total participants, each different hallway would be seen in combination with each different type of virtual human (including no virtual human), in each different possible order. Specifically, six participants were assigned to the six different possible presentation orders of the combinations (H1,A1), (H2,A2), (H3,A3), six to different presentation orders of (H1,A2), (H2,A3), (H3,A1), and six to different presentation orders of (H1,A3), (H2,A1), (H3,A2). When examining the data upon completion of the experiment, however, we noticed that due to an unfortunate oversight during the manual execution of the experiment, participant 11 had inadvertently been immersed in the condition (H1,A1) in the second block instead of (H3,A1), meaning that they saw H1 twice and didn't see H3, and participant 12 had been presented with (H2,A3), (H3,A2), (H1,A1) instead of (H2,A1), (H3,A2), (H1,A3). Therefore, although the virtual human conditions were distributed evenly across participants, some VH/hallway combinations were used more frequently, overall, than others.

**Figure 5:**
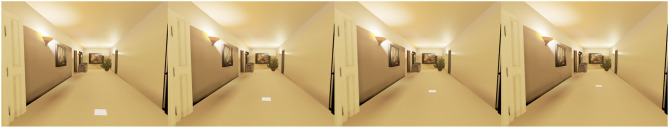
A forward-facing view of the four more distant targets in one of the three hallways. To see the nearest target, it would be necessary to look down slightly.

During each block of trials, the participant began by standing at a predefined location in the registered real and virtual environments, marked by some tape on the floor of our lab. Figure 1 shows what each of the hallway environments looked like from the “home base” position. After the participant had a chance to briefly look around (without moving from the home base position), their starting position was recorded using a key-press, a small white target was presented at a predefined location on the floor of the virtual hallway, and participants were asked to fixate on the target and when ready, to close their eyes, say “ready,” and walk to where they thought the target was. Figure 5 shows what the targets looked like. Upon hearing the word “ready,” the experimenter pressed a key to turn the display to black so that participants would not be able to see anything even if they did open their eyes. When the participant stopped, the experimenter used another key press to record their stopping location. As we had discovered, during pilot testing, that positional recording at the farthest distances could sometimes fail when the participant was facing away from their starting position because of the limitations in the range of the tracking system, we asked participants on each trial to turn around in place so that we could make a second recording of their ending position. When analyzing the data, we used information derived from the second recordings to infer the participant's ending position in the rare cases where the first recording failed. More details on this procedure are provided in the results section. With their eyes still closed, participants were then led on a circuitous path back to the home base position to start the next trial. Each block consisted of a total of 5 trials, in which targets were presented at distances of 8′, 10′, 12′, 14′, and 16′, in randomized order.

Following each block of trials, participants removed the HMD, filled out a SSQ survey, and enjoyed some water and a package of pretzels, cookies, or crackers. After the final block of trials, participants completed a brief presence questionnaire, in which they provided numeric answers on a scale from 1 to 7 to a total of 13 different questions intended to assess various aspects of their sense of presence in the virtual environment. These questions, provided in the Appendix A, were drawn from a combination of the Witmer-Singer IPQ (Witmer and Singer, 1998) and the Slater-Usoh-Steed presence questionnaire (Usoh et al., 2000). Finally, participants were asked four additional exit survey questions related to their impression of the relative realism of the different hallway environments, the realism of the virtual human, the strategy they used to arrive at the target square, and any suggestions they had for improving the virtual environment experience.

#### 3.1.5. Results

On eight of the 270 total trials, the outward facing ending position was recorded as (0, 0, 0) due to the inability of the HMD's sensors to see the light streams emanating from the Lighthouse tracking stations. These trials affected a total of 4 of our 18 participants. For each of those participants, we computed the median offset between the positions successfully recorded in the outward-facing and inward-facing directions on all of their other trials to derive an average “correction vector” that we then added to the inward-facing direction recorded at each point where the outward-facing position was unresolved, in order to infer the missing value(s). This procedure was used because the tracked position of the HMD moved in a systematic way when participants rotated in place, due to the HMD being located in front of the face while the axis of rotation was closer to being through the middle of the head. In every case that an outward-facing measure was invalid, an inward-facing measure was available. Additionally, on four out of the 270 trials, we discovered that only one ending position had been recorded in the data file, most likely due to experimenter error. In those cases, we interpreted the single recorded value as if it were a valid outward-facing value as this seemed the most likely occurrence and was also the most conservative action, given that the correction factors, when they were needed, would add several centimeters to the distance measured from the inward-facing orientation.

**Figure 6:**
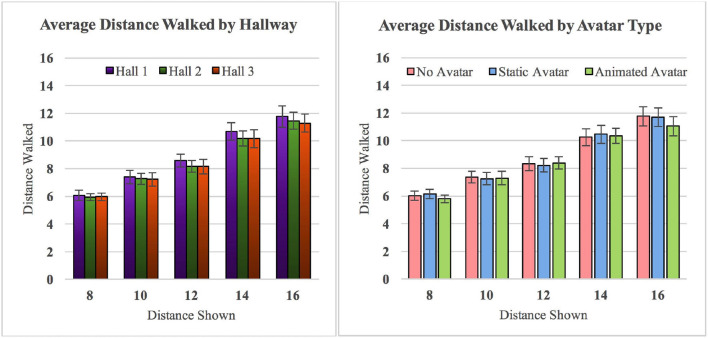
**(Left)** Average distance walked in each of the three different hallway environments, pooled over all three virtual human conditions. **(Right)** Average distance walked in each of the three different VH conditions, pooled over all three hallway environments. Error bars represent ±1 standard error (Paraiso and Interrante, 2017).

To analyze the results, we ran a three-way ANOVA (3-hallway × 3-VH_type × 5-distance_shown), using type III SS to account for the unbalanced data. We found a significant main effect of distance shown on distance walked [*F*_(4, 225)_ = 50.39, p = 0, $\eta_{p^{2}=0.473}$], but no significant main effect either of hallway environment [*F*_(2, 225)_ = 0.37, p = 0.6918, $\eta_{p}^{2}=0.0033$] or virtual human type [*F*_(2, 225)_ = 0.17, p = 0.8434, $\eta_{p}^{2}=0.0015$]. We also found no significant two-way or three-way interactions. Figure 6 shows plots of the average distance walked, for each distance shown, in each hallway environment and each virtual human condition.

**Figure 7:**
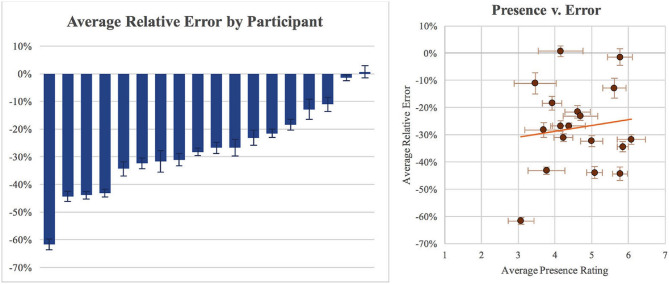
**(Left)** A plot of the average relative error in the distance judgments made by each participant, pooled over all hallway and agent conditions and ordered from greatest to lowest average error. **(Right)** A scatter plot, in which each disk represents the averaged data for one participant, showing little correlation between participants' subjective ratings of presence and the accuracy of their distance judgments in the virtual environment. Error bars represent ±1 standard error in each chart (Paraiso and Interrante, 2017).

Using a one-way ANOVA, we found significant differences in distance judgment accuracy between participants [*F*_(17, 252)_ = 43.361, p < 0.001, η^2^ = 0.758]. Note that we were unable to compute a multi-way ANOVA with participant as a factor because different people saw different combinations of agents and hallways. Among our 18 participants, two individuals had only negligible average error rates of 0.7% and –1.4%; at same time, one individual had a remarkably high average error rate of −61.6%. Between these two extremes, three participants had moderately high average relative errors in the range of −43.2 to 44.3%, while the remainder were in the range of −11 to 34%. Figure 7 (left) shows all of these results. Over all participants and all conditions, the average relative error in distance judgment accuracy was −27.35%.

Also using a one-way ANOVA to perform another sanity check, we found no significant impact of block order on error rates [*F*_(2, 267)_ = 1.10, p = 0.334, η^2^ = 0.0082]. This indicates that participants' overall task performance (pooled across all conditions) remained generally consistent over time.

Results from the SSQ surveys revealed no evidence of cybersickness.

Tabulating the results of the presence questionnaire, we found only moderate agreement with most questions, evidenced by an average Likert score of 4.63, after reversing the scores on the two questions whose scales ran from high to low rather than low to high. We also found no clear correlation between presence rating and distance judgment accuracy (r = 0.12), with the slope of the trendline being driven primarily by a single outlying participant. Figure 7 (right) illustrates the relevant data.

In the exit survey, in response to the question: “Did all three hallways seem equally realistic, or did one seem more realistic than the others?” we found a few participants with a preference for hallway 2, and a few who complained about hallway 3 and about the animated agent, but the majority of participants (10 of 18) reported that the hallways appeared equally realistic (8), or equally unrealistic (2).

Overall, participants were not impressed with the realism of the virtual human. In response to the question: “Does the virtual person appear realistic/human-like or did it feel more un-human?” the majority (11 out of 18) directly replied that the VH seemed unrealistic or un-human, and six of these, in elaboration, explicitly complained the movement being unnatural, jerky, glitchy, or “obviously animated.” Only three of the 18 participants gave an unqualified response of “realistic” or “human-like” to this question; three more gave qualified responses (e.g., “somewhat,” or “realistic with the exception of…”), which might be explained by politeness. Additionally, one participant remarked that the VH felt human-like only because it resembled the experimenter; they said that they were not sure if they would have thought the same if the virtual human had been a stranger.

When asked “What strategy did you use to arrive at the target square?” 10 out of 18 participants responded with some variation on estimating the number of steps that would be required to arrive at the target. The other 8 of 10 reported using a more holistic strategy, as in “I tried to remember the scene and imagine it when my eyes were closed,” or “I looked at the visual landmarks of the hallway and thought about passing them.” Taking a pre-estimated number of steps can be a problematic strategy for indicating distance in VR because people tend to unconsciously take smaller steps while wearing an HMD than in the real world (Mohler et al., 2007; Phillips et al., 2010).

For the open-ended question: “Is there anything else you can tell us to help us improve the virtual environment experience?” 8 of 18 participants had no response or just said “no.” However, three participants complained about the lighting of the virtual model, and two remarked on issues related to distance perception: one said that they felt too short in the virtual environment, and the other reported that the depth seemed “off…as though the hallway was not as deep as it was visually.” Also, one participant said that the headset felt heavy. In addition, three participants remarked on factors related to stopping before they reached the target: one reported a fear of walking into things, one said that the automatic appearance of the Vive boundary grid (which they said could be noticed even when their eyes were closed) was distracting, and one participant told us: “When I got near the point, the display went blue. Don't do that. Then you kind of know when to stop,” suggesting that they were mis-interpreting the change in intensity seen through their eyelids when the boundary grid appeared as an indication that the target had been reached.

#### 3.1.6. Discussion

We were reassured to find that results analysis seemed to confirm the robustness of the general design of our experiment, in particular the choice to expose participants to each of the different virtual human conditions in a different virtual environment, which we felt was important to minimize the likelihood of carry-over effects between blocks. Although we had taken care to construct the three hallway environments to be as structurally similar as possible while at the same time clearly representing different places, the possibility remained that some of the differing characteristics might potentially facilitate or hinder distance judgment accuracy in unforeseen ways in some environments relative to others. For example, rooms with darker colored walls have been reported to appear smaller than similar-sized rooms with lighter colored walls (Stamps, 2010), and Witt et al. (2007) report a significant impact of the environmental context beyond a target on the accuracy of peoples' judgments of their distance to the target, in real world scenes. Fortunately, such complications did not appear to significantly affect distances walked in this experiment.

The most notable outcome from this experiment is the lack of any indication of a significant impact of the presence or absence of our replica virtual human model on the accuracy of participants' egocentric distance judgments in the tested virtual environments. Although we can obviously not claim to have proven the null hypothesis, our finding of a consistent, significant effect of target distance on distance walked supports the face validity of the experimental procedure and bolsters the likelihood that our other results are also well-founded. There are many possible explanations for this disappointing outcome. It could be that people do not tend to use the heights of other inhabitants in a shared virtual environment as absolute indicators of the scale of the space, or it could be that autonomous agents have little influence on peoples' sense of scale in VR. It is also possible that certain characteristics of the particular virtual human model we used caused it to be discounted as a reliable indicator of scale, possibly due to visual or behavioral artifacts that reduced peoples' trust in its robustness as a faithful representation of the depicted person. Alternatively, it could be that the absolute size cues provided even by a fully realistic model of a familiar known person might be insufficient to overcome other more fundamental factors underlying the distance underestimation phenomenon.

It is informative to observe that participants in this experiment underestimated egocentric distances, on average, by a considerable amount (~25%). This is slightly more than the 15–20% underestimation observed in the HTC Vive by Kelly et al. (2017), and moderately more than the 15% underestimation observed by Creem-Regehr et al. (2015) using the Oculus Rift. Several limitations of our implementation may have contributed to these increased errors. Most notably, participants had a relatively low opinion of the visual realism of the virtual environment, and they also reported relatively low ratings of experiential realism, or presence. As can be seen in Figure 1, the virtual hallway models used in this experiment exhibited notable inconsistencies in the physical plausibility of the lighting appearance, and there were significant artifacts in both the static appearance and dynamic motion of the virtual human model. Previous work in our lab has suggested that even a little unreality in an otherwise viscerally realistic VR scenario can have significant consequences for distance judgment accuracy (Phillips and Interrante, 2011). Other shortcomings include: the problematic appearance of the boundary grid on the trials in which participants walked too close to the limits of the tracked area, the inadvertent slightly uneven distribution of stimulus conditions among participants, and the necessity of using an approximate correction factor to infer several pieces of missing data. While we doubt that any of these latter irregularities played a significant role in shaping our qualitative findings, and we were able to verify that there was no change in our findings of significant effects (or lack thereof) when each of the 13 corrected data points was instead excluded from the statistical analyses, without further experimental work it is not possible to definitely rule out an impact of any of the other noted issues.

### 3.2. Second Experiment

Our first experiment found that adding a right-sized virtual 3D replica of a familiar person to an unfamiliar (i.e., never-before-experienced in real life) virtual environment had no positive impact on the accuracy of peoples' judgments of egocentric distances in a context in which distances were being underestimated. These results raised several questions that we sought to address in a followup study. First, and most importantly, we wondered: does the size of a third-person virtual human model have any potential at all to affect participants' judgments of egocentric distances in a shared virtual space? Although adding a right-sized virtual human had no positive effect, could adding a wrong-sized virtual human have a negative effect? This knowledge could inform how careful we need to be in selecting virtual human models if we want to proceed with using them as entourage elements. Secondly, we were somewhat concerned by the fact that the majority of the participants in our first experiment had reported relatively low levels of visual and experiential realism, and we wondered if that might somehow have played a role in our findings, possibly contributing to the slightly higher overall average amount of distance underestimation we had observed, relative to the findings reported in related prior work, or possibly even having a dampening effect on the potential for any positive impact of adding a virtual human agent to the environment. To further address other shortcomings we had identified in our first study, we also took steps to avoid the possibility that participants could walk beyond the bounds of our tracked area by using a different positional measuring system, and we decided to re-introduce a real world blind walking task to better control for any potential individual differences in general blind walking task performance, independent of the virtual environment condition. In all, our second experiment aimed to further explore the general potential of virtual human entourage elements to influence people's spatial understanding of a virtual indoor environment, specifically by assessing the impact of differently-sized virtual human replica models on participants' egocentric distance judgments under more nearly photorealistic modeling and rendering conditions.

#### 3.2.1. Method

While our first experiment had manipulated the *behavior* of the replica virtual human model between conditions (static vs. animated), with the no-agent condition as a control, our second experiment manipulated the *size* of the replica virtual human model, using a between-subjects design to avoid drawing participants' attention to the size manipulation. Three subgroups of participants were each exposed to one of three different conditions of a virtual human replica entourage element: actual-sized (160 cm), 20% smaller than the actual size (128 cm), or 20% larger than the actual size (192 cm). Participants used blind walking to estimate egocentric distances in a virtual hallway model within which the virtual agent was standing, and we also asked participants to perform blind walking trials in a real world environment as a pre-test in order to be able to normalize for individual differences in overall blind walking distance estimation performance.

#### 3.2.2. Participants

We recruited a total of 36 participants (22 male, 14 female, ages 16–33, μ = 21.6 ±4.2) from our local university community using email lists and posted flyers. Each participant was arbitrarily assigned to one of the three different virtual human size conditions, resulting in a total of 12 participants in each subgroup. All participants gave written informed consent, and received modest monetary compensation in the form of a $10 Amazon gift card. The experiment was approved by our University's Institutional Review Board and was conducted in accordance with the ethical principles outlined in the Declaration of Helsinki.

#### 3.2.3. Materials

**Figure 8:**
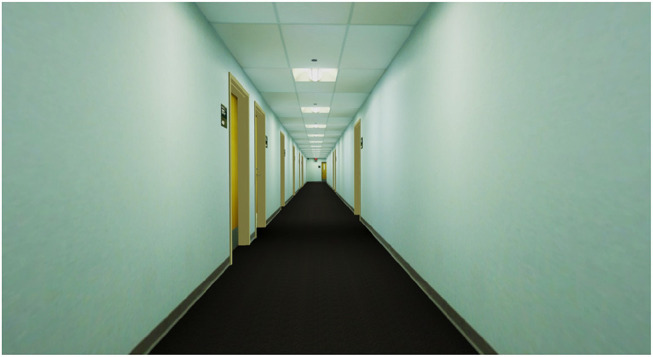
The photo-realistic hallway environment used in the second experiment.

The virtual environment we used in our second experiment was a nearly photo-realistic 3D replica model of a restricted-access hallway on our University campus that was created by a collaborator from the Department of Architecture at our University. The geometry was built using the SketchUp toolkit to exactly match the measurements of an existing real space and all of the major elements in the model, with the exception of the carpet, were texture-mapped using photographs taken in the corresponding real world environment. Figure 8 shows a screenshot of the virtual hallway model.

For the virtual human model, we obtained a high-quality 3D body scan of the first author, who would be running the second experiment, from Me3D^2^, a commercial 3D scanning company that has a 360° photo booth in the Mall of America. We then scaled the model using Maya 2018 to match the width and the height of the experimenter, ensuring that our model was exactly the right size. Next, we imported the model into Mixamo for rigging, so that we could adjust the model's posture to have a natural pose. As in the previous experiment, the experimenter wore the same outfit when conducting the experiment.

**Figure 9:**
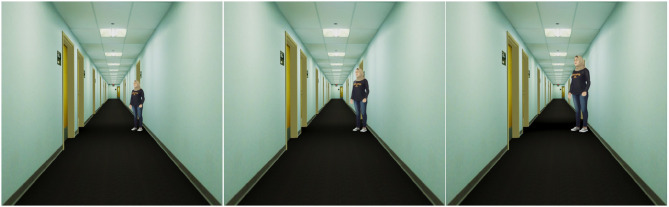
The three differently sized virtual human models, seen from the same vantage point in the virtual hallway.

In each of the three VH conditions, the corresponding small, natural, or large sized 3D virtual human model was placed slightly to the side of the virtual hallway and moderately far behind the most distant of the targets that would be used in the blind-walking trials. Figure 9 shows what the three different sized VH models looked like from the same point in the virtual hallway environment.

The second experiment was conducted in the same laboratory space as the first experiment, an approximately 30' × 29' room. Participants viewed the virtual environment using an Oculus Rift CV1 head-mounted display, which, like the HTC Vive in our first experiment, uses two separate OLED displays to present separate 1080 × 1200 images to each of the observer's eyes, with partial stereo overlap. The virtual environment was rendered using the Unreal Engine on an MSI GT72 Dominator Pro-445 gaming laptop with an 8 GB NVIDIA Geforce GTX 980M graphics card, and a 2.80 GHz Intel Core i7 4980 HQ processor with 32 GB of memory and a 512 GB SSD. The laptop was set on a small table in the center of the room, and was connected to the HMD using 6′ USB 3.0 and high-speed HDMI extension cables, allowing the user to freely traverse a distance of well over 24′. The position and orientation of the user's head was tracked in real time within the full area of our lab space using 16 Vicon Vero 2.2 cameras. To accomplish this, we designed and built a removable 3D-printed attachment for the HMD that provided a four-pronged mounting platform for the retro-reflective tracking marker balls.

#### 3.2.4. Procedure

Each of the participants was greeted at the door of our lab by the experimenter who would be represented by the virtual human model in the VR environment. They took the same visual acuity and stereo vision ability tests used in our first experiment, then read the instructions explaining the experimental procedure and gave their informed consent to participate by signing a consent form. Three prospective participants were disqualified from participating due to failing the visual acuity test, highlighting the importance of performing such testing in situations where it is not feasible or not comfortable to wear corrective lenses inside the HMD. All prospective participants passed the stereo vision ability test. Before beginning the experiment, participants filled out a short survey providing basic demographic information. Once these initial steps were complete, the experimental testing began.

**Figure 10:**
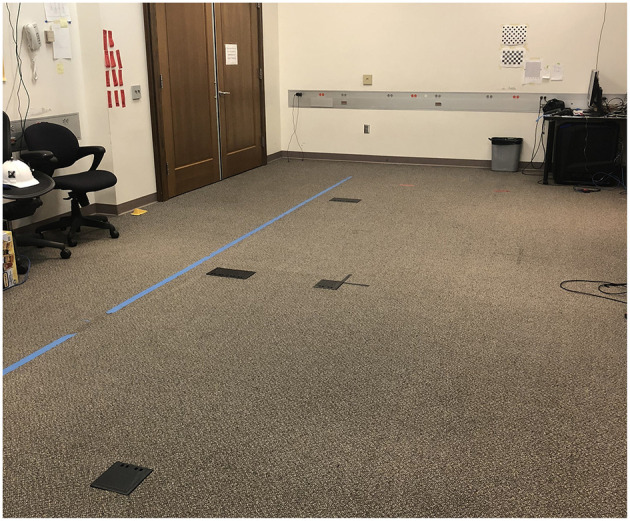
Real world environment that we used in the second experiment.

Participants started out by making a total of 4 distance judgments in the physical lab space, using direct blind walking. Figure 10 shows the what this space looks like. The distances used for the real world trials were 7′, 11′, 13′, and 17′, and they were presented in randomized order. Each trial began at the same, pre-defined home base position, which was marked by a large piece of tape on the floor. Participants wore a white hard hat with Vicon tracking markers attached to enable easy recording of their starting and ending locations, and they used a black sleep mask to cover their eyes. At the start of each trial, the participant closed their eyes and wore the blindfold while the experimenter placed a piece of red tape at the target location. Small sewing pins were discretely hidden in the carpet to help the experimenter quickly place the target mark at the correct distance for each trial. After the experimenter had finished placing the target they stood behind the participant, recorded their position using a key press, then directed the participant to take off the blindfold, take visual aim at the target, and then close their eyes, replace the blindfold, and walk to where they think the target is. After the participant stopped walking, the experimenter used another key press to record their ending location. With their eyes still closed, participants were led back to the home-base position via a circuitous path to start the next trial. Participants did not get any feedback about their performance at any time, and the tape marks were placed so that the walking path was well to the side of the plastic electrical box covers visible in Figure 10.

**Figure 11:**
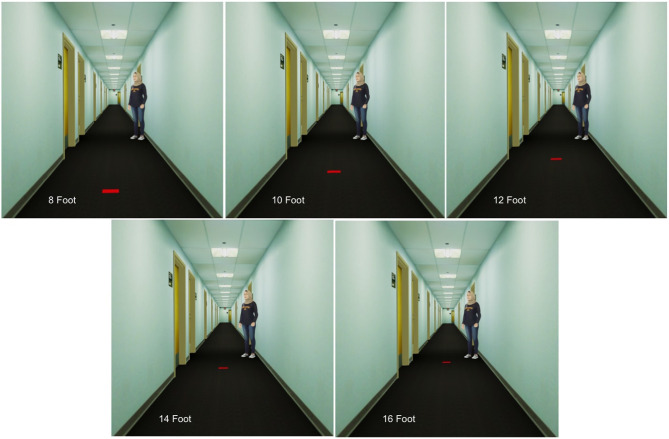
Participants' view of the naturally-sized virtual human in the virtual hallway with targets presented at distances of 8′, 10′, 12′, 14′, and 16′.

In the second block of trials, participants made a total of 7 distance judgments in the VR using blind walking. The first two distances shown were 9′ and 15′ (presented in randomized order), and the data from those trials was treated as training and discarded. The next five distances shown were 8′, 10′, 12′, 14′, and 16′, also presented in randomized order. As in the real world trials, participants began by standing at or near a predefined, unmarked home-base location, which was registered to the same position in the real and virtual environments. Figure 11 shows what the participant saw, from the home-base position, for each marker distance. At the start of each trial, the virtual environment was revealed in the HMD. After participants were given the opportunity to briefly look around, without moving from the home-base position, a small red target was presented on the floor of the virtual hallway and the participant's starting position was recorded. Just as in the real world trials, participants were instructed to take visual aim at the target and then, when ready, close their eyes, say “ready,” and walk to where they think the target is. Upon hearing the word “ready,” the experimenter pressed a key to turn the display to black so that participants would not be able to see anything even if they were to accidentally open their eyes. After the participant had stopped walking and said “done,” the experimenter used another key press to record their ending location. With their eyes still closed and the display still black, participants were led, via a circuitous path, back to the home-base position to start the next trial.

After completing the VR trials, participants took off the HMD and filled out a brief questionnaire, in which they answered 11 questions assessing various aspects of their sense of presence in the virtual environment using a 7-point scale. These questions, provided in Appendix B, were adapted from a combination of the Witmer and Singer (1998) and the Slater-Usoh-Steed presence questionnaires (Usoh et al., 2000).

Finally, participants were asked seven additional questions to collect their impressions of the realism of the virtual hallway environment, the realism of the virtual human model, the strategy they used to arrive at the target, their sense of the size of the virtual human and the virtual hallway, and any suggestions they had for improving the virtual environment experience.

#### 3.2.5. Results

**Figure 12:**
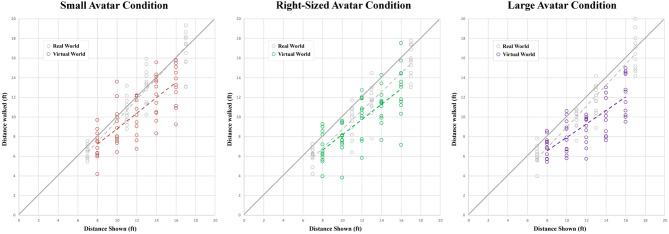
Scatter plots of the distances walked by each participant in each of the virtual human size conditions.

**Figure 13:**
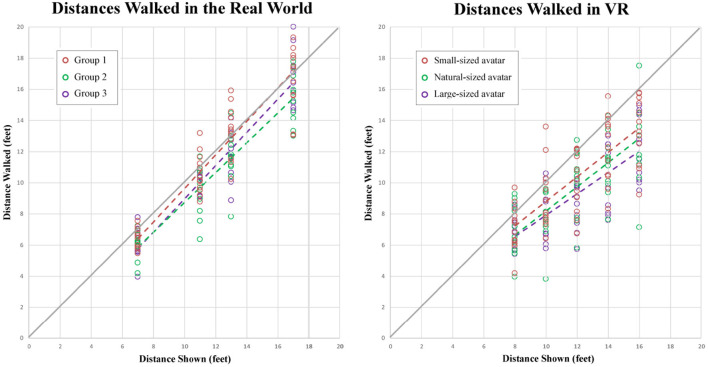
Scatter plots of the distances walked by each participant in the real and virtual environments.

Figures 12 and 13 show different views of the results from our second experiment. In Figure 12, each graph compares the distances walked in the real world (gray) and the virtual environment (colored) by each participant in each of the participant groups. In Figure 13, these same results are arranged to facilitate between-group comparisons in the real (left) and virtual (right) conditions.

We used a two-way Anova (3-VH_size × 5-distance_shown) with replication (12 participants in each group) to analyze the results from the VR trials. As expected, we found a significant main effect of distance shown on distance walked [*F*_(4, 165)_ = 55.38, p = 0, $\eta_{p}^{2}=0.573$]. Tukey *post-hoc* tests found that all pairwise differences were significant. Interestingly, the same two-way Anova also found a significant main effect of the size of the virtual human model on the distance walked [*F*_(2, 165)_ = 4.996, p = 0.0078, $\eta_{p}^{2}=0.0571$]. Tukey *post-hoc* tests found that group 3 (who saw the larger virtual human model) walked significantly shorter on average than group 1 (who saw the smaller virtual human model), but no other pairwise differences were significant. Overall, distances were underestimated by an average of 21% by participants in the larger-sized virtual human condition, by an average of 18% by participants in the normal-sized virtual human condition, and by an average of 13% by participants in the smaller-sized virtual human condition. The overall average relative error in distance estimation across all VR conditions was -17.8%, a number that is comparable to results found in related work by other groups.

We also used a two-way Anova (3-participant_group × 4 distance_shown) with replication (12 participants in each group) to analyze the results from the real world trials. All of the real world trials were done prior to any VR exposure, and were intended to serve as a potential baseline control for possible individual differences in blind walking task performance, which might otherwise have an inscrutable effect on the between-subjects comparison of distances walked in the different virtual human conditions. As expected, we found a significant main effect of distance shown on distance walked [*F*_(3, 132)_ = 300.87, p = 0, $\eta_{p}^{2}=0.872$], and Tukey *post-hoc* tests found that all pairwise differences were significant. The same two-way Anova also found a significant main effect of participant group on distance walked [*F*_(2, 132)_ = 6.948, p = 0.0013, $\eta_{p}^{2}=0.0952$]. Tukey *post-hoc* tests found that group 2 (who would later see the natural-sized virtual human model) walked significantly shorter on average than group 1 (who would later see the smaller-sized model), but no other pairwise differences were significant. Real world distances were underestimated by an average of 11.8% by participants in group 2 (who would go on to experience the normal-sized virtual human model), by an average of 8.8% by participants in group 3 (who would go on to experience the larger-sized model), and by only 2.9% by participants in group 1 (who would go on to experience the smaller-sized model). We believe that awareness of individual differences is particularly important when interpreting distance underestimation errors between conditions when a between-subjects experimental design has been used.

Seeking a more integrated understanding of our data, we used a third two-way Anova (3-group × 2-environments) to analyze the potential effect of these two factors on the average relative error in participants' distance judgments, computed as (distance_walked - distance_shown)/distance_shown) and averaged, for each participant, over each of the 4 or 5 different distances shown in the real and virtual environments, respectively. In this Anova, we found a significant main effect of environment [*F*_(1, 66)_ = 14.487, p = 0.00031, $\eta_{p}^{2}=0.180$]–people made greater errors in VR than in the real world – and also a significant main effect of group [*F*_(2, 66)_ = 3.448, p = 0.0377, $\eta_{p}^{2}=0.0946$]. Tukey *post-hoc* tests found that participants in group 1 made smaller errors than participants in groups 2 and 3 at α = 0.10, but not at α = 0.05. No other pairwise differences were significant.

In the exit survey for our second experiment, a substantial minority of participants (8 of 36) admitted to using some sort of step-counting-related strategy to arrive at the target mark, despite our best efforts to more explicitly request, in the written instructions, that they avoid using any artificial strategy, including counting steps, when walking the perceived distance. While we were somewhat encouraged to know that more participants followed our instructions this time, the stubborn persistence of the step-counting strategy is notable, as is the fact that telling people not to do it seems insufficient to eliminate the practice.

In contrast with our first experiment, most of the participants in our second experiment expressed qualified satisfaction, in our exit survey, with the realism of the virtual hallway and the virtual human. Suggestions for improving the realism of the environment centered on factors such as improving the lighting and increasing the quality of the display; suggestions for improvements to the virtual human primarily focused on the need for increased detail, while also mentioning a desire for the virtual human to move and respond to the participant. Interestingly, despite the clearly more positive overall tone of participants' open-ended comments, we did not see a significant increase in the overall numeric ratings in the presence questionnaire, compared to our first experiment. This serves as a reminder that such scores are notoriously problematic to compare between experiments, as the subjective baseline for the scoring is unlikely to be consistent (Slater, 2004).

The majority of participants (22 of 36), when directly queried about the naturalness of the hallway's size, reported that it seemed correctly proportioned. However a sizeable minority (10 of 36) reported that it seemed too narrow, or (3 of 36) otherwise ill-proportioned. Only one participant explicitly mentioned that the virtual human had an influence on their impression of the appropriateness of the hallway's size. When directly queried about the agent's size, 9 of the 12 participants who experienced the larger agent condition recognized that the model was too tall, but only 3 of the 12 participants who experienced the smaller agent condition recognized that the agent was smaller than normal. Of the 12 participants who experienced the unmodified size condition, 11 reported that the agent seemed correctly-sized; one remarked that the agent felt somehow too small.

#### 3.2.6. Discussion

The primary goal of our second experiment was to further elucidate the potential impact of virtual human entourage elements on distance perception in VR. While Figure 13 seems to show a trend toward greater distance underestimation in the presence of larger-than-life virtual human agents, and a statistical analysis of the VR data shows significant differences in distances walked between the larger and smaller virtual human conditions, a careful consideration of the baseline real-world data suggests that individual differences in blind walking task performance may be partially responsible for those results.

The most interesting result from our second experiment therefore is the relative lack of effect of the metric size of the virtual human model on participant's distance judgments in VR – not only were participants nearly as equally accepting of the “naturalness” of a 20% downsized virtual replica of a familiar person as of a right-sized model, but even when the virtual human model was recognizably over-sized, participants' distance judgment accuracy was not clearly significantly affected as a result. It therefore seems reasonable to conclude that, at least in the context of our present experiment, participants' sense of the scale of a realistic virtual environment is not significantly affected by moderate distortions to the size of a virtual human entourage element, even when the virtual human model represents a familiar person whose true height is known from direct experience. This suggests that people may not tend to use the heights of other people in a shared space to calibrate their perception of that space, at least not in a fine-grained way. Although disappointing, this finding is consistent with the previous results of Linkenauger et al. (2013) who found no effect on object size perception of scale changes to other peoples' hands despite a significant own-hand size effect.

A secondary goal of our second experiment was to try to address the significant shortcomings in the visual quality of the virtual hallway and virtual human models noted by participants in our first experiment and to avoid some of the glitches that had arisen in our experimental procedure, with an eye toward potentially improving distance estimation performance overall, to at least match the average levels reported in related work. Distance underestimation errors were in fact slightly less severe, on average, in our second experiment than in our first. However, we cannot robustly claim that the improved visual and experiential quality of our second experiment was solely responsible for the more modest distance underestimation errors we observed, in comparison to our first experiment, because there were also other differences between the first and second experiments that could have had an impact, including a change of headset (from Vive to Rift) and a change of tracking system (from LightHouse to Vicon). Buck et al. (2018) found that participants tended to underestimate distances more when using an HTC Vive than an Oculus Rift display. A thorough examination of additional factors potentially influencing distance estimation judgments made via blind walking is outside the scope of this paper, but if distance estimation continues to be a topic of interest in VR research, future work might more extensively consider a wider range of other factors, such as obvious rendering errors and other procedural glitches, in particular those that might evoke a sense of decreased experiential realism or heightened breaks in presence, as well as practical concerns like an over-cluttered lab space, that might unanticipatedly degrade performance on blind walking tasks.

The moving bar of participant's expectations of realism serve as a reminder that we still have a fairly long way to go before basic architectural design- focused immersive VR experiences will feel so compellingly realistic as to be nearly equivalent to a comparable real-world experience. We find it notable that, in experiment 1, participants' average numerical ratings of the realism of the virtual environment (e.g., in response to questions such as: “How real did the virtual world seem to you?”) were nearly equivalent (at 4.28) to participants' ratings to similar questions in experiment 2, where the average ratings associated with the questions “How real did the appearance of the virtual environment look to you?” and “How real did the virtual environment experience feel to you?” were 4.53 and 4.67, respectively. This reinforces the value of asking free-response questions, despite the difficulty of their analysis, and re-emphasizes the observation made many years ago by Mel Slater about how caution must be exercised when interpreting Likert ratings of things like “realism” or “presence” in VR, particularly when making comparisons between experiments (Slater and Garau, 2007).

## 4. Conclusions and Future Work

The main take-home message from our two reported experiments is that exact-scale virtual human entourage elements do not appear to provide an easy answer to the long-studied problem of distance underestimation in HMD-based VR. Specifically, we found little evidence to support our hope that populating virtual environments with static or dynamic virtual human models will help people to more accurately assess egocentric distances in those environments. Conversely, we found that participants were surprisingly insensitive to moderate inaccuracies in the metric size of a virtual human that had been modeled after a familiar known person, painting a rather discouraging picture of the potential promise of virtual human entourage elements to act as reliable “rulers” by which people might calibrate their perception of size and distance in an unfamiliar building model.

We are nevertheless still encouraged to pursue efforts to bring virtual architectural models “to life” through the use of autonomous intelligent agents; even if these virtual entourage elements do not lead to more accurate judgments of egocentric distances in VR, they remain likely to be useful in conveying a more accurate subjective sense of how a space will feel under typical use conditions. Especially in cases involving the design of large public buildings, such as schools, libraries, and hospitals, accurately representing large shared spaces in a representatively inhabited state could be equally as important and informative to peoples' subjective sense of the suitability of the design as providing an accurate impression of the metric dimensions of the space. Many future challenges await in that effort, from appropriately modeling both coarse and fine details of virtual agents' appearance and interactive behaviors to avoiding the uncanny valley.

## Ethics Statement

This study was carried out in accordance with the University of Minnesota's policies on the Responsible Conduct of Research. The study protocol was approved by the Institutional Review Board of the University of Minnesota. All participants gave written informed consent.

## Author Contributions

VI designed and planned the experiments in consultation with KP and SA. KP did all of the virtual human and environment modeling, software implementation, and data collection for the first experiment. SA did all of the virtual human modeling, software implementation, and data collection for the second experiment. VI assisted with the data analysis and contributed to the interpretation of the data and discussion. VI and SA took the lead in writing the manuscript.

### Conflict of Interest Statement

The authors declare that the research was conducted in the absence of any commercial or financial relationships that could be construed as a potential conflict of interest.

## Footnotes

^1^https://gumroad.com/l/uPXbQ

^2^https://me3dstore.com

## A. Appendix

Presence questions for first experiment (based on the Universit du Qubec en Outaouais Cyberpsychology Lab's revision [http://w3.uqo.ca/cyberpsy/docs/qaires/pres/PQ_va.pdf] of Witmer and Singer (1998) Presence Questionnaire and the Slater-Usoh-Steed Usoh et al. (2000) Presence Questionnaire):

1. In the computer generated world, I had a sense of “being there” (1 = not at all; 7 = very much).
2. When you think back to the experience, do you think of the virtual environment more as images that you saw or more as somewhere that you visited? (1 = images; 7 = felt like I vis-ited).
3. How aware were you of the real world surrounding while navigating in the virtual world (i.e., sounds, room temperature, other people, etc.)? (1 = Not aware at all; 7 = extremely aware) [reversed for scoring].
4. To what extent were there times during the experience when the virtual environment was the reality for you? (1 = none; 7 = always).
5. How real did the virtual world seem to you? (1 = not real at all; 7 = completely real).
6. How much did your experience in the virtual environment seem consistent with your real world experience? (1 = not consistent; 7 = very consistent).
7. How natural did your interactions with the environment seem? (1 = unnatural; 7 = very natural).
8. How compelling was your sense of objects moving through space? (1 = not at all; 7 = very compelling).
9. How completely were you able to actively survey or search the environment using vision? (1 = not at all; 7 = completely).
10. How compelling was your sense of moving around inside the virtual environment? (1 = not at all; 7 = very compelling).
11. How quickly did you adjust to the virtual environment experience? (1 = not at all; 7 = quickly adjusted).
12. How proficient in moving and interacting with the virtual environment did you feel at the end of the experience? (1 = not reasonably; 7 = very proficient).
13. How much did the visual display quality interfere or distract you from performing as-signed tasks or required activities? (1 = not at all interfered; 7 = interfered a lot) [reversed for scoring].

## B. Appendix

Presence questions for second experiment (based on the Universit du Qubec en Outaouais Cyberpsychology Lab's revision [http://w3.uqo.ca/cyberpsy/docs/qaires/pres/PQ_va.pdf] of Witmer and Singer (1998) Presence Questionnaire and the Slater-Usoh-Steed Usoh et al. (2000) Presence Questionnaire):

1. In the computer generated world, I had a sense of “being there” (1 = not at all; 7 = Extremely much).
2. When you think back to the experience, do you think of the virtual environment more as images that you saw or more as somewhere that you visited? (1 = Definitely as images I saw; 7 = Definitely as somewhere I visited).
3. How aware were you of the real world surrounding while navigating in the virtual world? (i.e., extraneous sounds, fear of colliding with obstacles in the room, presence of other people in the lab space, etc.)? (1 = Extremely aware; 7 = Not at all aware) [reversed for scoring].
4. How much were you affected by the real world surrounding while navigating in the virtual world? (i.e., extraneous sounds, fear of colliding with obstacles in the room, presence of other people in the lab space, etc.)? (1 = Extremely affected; 7 = Not at all affected).
5. To what extent were there times during the experience when you were so completely captivated by the virtual environment that it was reality for you? (1 = Never; 7 = Always).
6. How real did the appearance of the virtual environment look to you? (1 = Not at all realistic; 7 = Identical to the real world).
7. How real did the virtual environment experience feel to you? (1 = Not at all realistic; 7 = Identical to the real world).
8. To what extent did your experience in the virtual environment seem consistent with your real world experience? (1 = Not at all consistent; 7 = Perfectly consistent).
9. How natural did your interactions with the environment seem? (1 = Completely unnatural; 7 = Completely natural).
10. How much did the VR technology (e.g., visual display quality, cords tethering the HMD to the computer, weight/discomfort of the HMD, etc.) interfere or distract you from performing assigned tasks or required activities in a natural way? (1 = Completely; 7 = Not at all).
11. How quickly did you adjust to the virtual environment experience? (1 = Never; 7 = Immediately).
